# Phenotypic and genotypic characterization of antimicrobial resistance and virulence profiles of *Salmonella enterica* serotypes isolated from necropsied horses in Kentucky

**DOI:** 10.1128/spectrum.02501-24

**Published:** 2025-01-23

**Authors:** Ajran Kabir, William G. Kelley, Cheyenne Glover, Erdal Erol, Yosra A. Helmy

**Affiliations:** 1 Department of Veterinary Science, Martin-Gatton College of Agriculture, Food, and Environment, University of Kentucky, Lexington, Kentucky, USA; 2 College of Veterinary Medicine, Lincoln Memorial University, Harrogate, Tennessee, USA; 3 Veterinary Diagnostic Laboratory, Martin-Gatton College of Agriculture, Food, and Environment, University of Kentucky, Lexington, Kentucky, USA; Innovations Therapeutiques et Resistances, Toulouse, France

**Keywords:** *Salmonella*, biofilm, antimicrobial resistance, MDR, resistance genes, horses, motility

## Abstract

*Salmonella* is a foodborne pathogen that poses a significant threat to global public health. It affects several animal species, including horses. *Salmonella* infections in horses can be either asymptomatic or cause severe clinical illness. Infections caused by *Salmonella* are presently controlled with antibiotics. Due to the formation of biofilms and the emergence of antimicrobial resistance, the treatment has become more complicated. Our study focused on investigating the prevalence of *Salmonella enterica* in necropsied horses, assessing the capability for biofilm formation, and motility, determining the phenotypic and genotypic profiles of antibiotic resistance, and detecting virulence genes. A total of 2,182 necropsied horses were tested for the presence of *Salmonella*. Intestinal samples were enriched in selenite broth and cultured on hektoen and eosin methylene blue agar plates, whereas other samples were directly cultured on aforementioned plates. Confirmation of the serotypes was performed according to the Kauffmann–White–Le Minor Scheme followed by biofilm formation screening using crystal violet assay. The resistance profile of the isolates was determined by broth microdilution assay using the Sensititre️ Vet (Equine EQUIN2F). The genotypic antimicrobial resistance (AMR) and virulence profiles were detected using polymerase chain reaction (PCR). The overall prevalence of *Salmonella* was 1.19% (26/2182), with 11 different serotypes identified. *Salmonella* Typhimurium was the most prevalent serotype with 19.2% prevalence. All of the isolates were identified as biofilm producers and motile. Virulence genes related to invasion (*invA, hilA, mgtC,* and *spiA*), biofilm formation (*csgA* and *csgB*), and motility (*filA, motA, flgG, figG, flgH, fimC, fimD,* and *fimH*) of *Salmonella* were detected among 100% of the isolates. An overall 11.4% of the isolates were identified as multidrug-resistant (MDR), with resistance to gentamicin, amikacin, ampicillin, ceftazidime, ceftiofur, chloramphenicol, and trimethoprim/sulfamethoxazole. We found that beta-lactamase-producing genes *bla_TEM_, bla_CTXM_*, and *bla_SHV2_* were identified in 11.5% of the isolates, while only 3.8% carried the *bla_OXA-9_* gene. The presence of MDR pathogenic *Salmonella* in horses is alarming for human and animal health, especially when they have a high affinity for forming biofilm. Our study found horses as potential sources of pathogenic *Salmonella* transmission to humans. Thus, it is important to perform continuous monitoring and surveillance studies to track the source of infection and develop preventive measures.

**IMPORTANCE:**

This study focuses on understanding how *Salmonella*, specifically isolated from horses, can resist antibiotics and cause disease. *Salmonella* is a well-known foodborne pathogen that can pose risks not only to animals but also to humans. By studying the bacteria from necropsied horses, the research aims to uncover how certain *Salmonella* strains develop resistance to antibiotics and which genetic factors make them more dangerous. In addition to antibiotic resistance, the research explores the biofilm-forming ability of these strains, which enhances their survival in harsh environments. The study also investigates their motility, a factor that contributes to the spread of infection. The findings can improve treatment strategies for horses and help prevent the transmission of resistant bacteria to other animals as well as humans. Ultimately, the research could contribute to better management of antibiotic resistance in both veterinary and public health contexts, helping to safeguard animal welfare and public health.

## INTRODUCTION

*Salmonella enterica* is a Gram-negative bacterium within the *Enterobacteriaceae* family and is one of the four primary contributors to diarrheal diseases worldwide (1). It is a prominent foodborne pathogen that can affect a wide range of hosts, including equines, and is also listed as a significant cause of illness and death by the World Health Organization (WHO) (2, 3). To date, more than 2600 *Salmonella* serotypes have been identified, all of which have the potential to cause cross-infections between animals and humans (3, 4) and most of them have been reported as the cause of significant economic losses globally (5).

Salmonellosis in equines is mainly associated with nosocomial infections along with on-farm contamination (6). Although subclinical infection and shedding of *Salmonella* are more common in horses, clinical infections can cause enterocolitis with acute severe diarrhea and protein-losing enteropathy (7). It was reported that the prevalence of subclinical *Salmonella* ranges between 1% and 2% in healthy horses (8–10); however, this number can increase up to 9%–13% in hospitalized horses suffering from colic or gastrointestinal symptoms (11). In addition, a significantly high mortality rate in equine veterinary hospitals (38%–44%) was associated with *Salmonella* infections (2). The most commonly isolated *Salmonella* serotypes in horses include *S. enterica* serovars Typhimurium, Newport, Javiana, Braenderup, Anatum, Infantis, Muenchen, and Mbandaka (5, 12–14). The presence of these human-associated serotypes in horses indicates that equines may act as a potential source of infection for other hosts, including humans (15, 16).

*Salmonella* transmission in horses primarily occurs via the fecal–oral route, either from contaminated food and environments or direct contact with other infected horses (5). Fecal shedding of *Salmonella* from horses contaminates the surrounding environment, and its ability to survive in damp environments poses a potential threat to a wide range of hosts, including humans (7, 17–19). The role of horses as working animals, livestock, and pets increases the chance of *Salmonella* transmission to humans (20–22). According to the CDC, *Salmonella* infections in humans contribute to around 1.35 million illnesses, 26,500 hospitalizations, and 420 fatalities each year in the US alone (1). Infections in humans cause self-limiting gastroenteritis. However, persistent non-treated infections may lead to both gallbladder and colon cancer (23).

The pathogenicity of *Salmonella* depends on several virulence factors that can lead to serious infections in the host (5, 24). The pathogenesis of *Salmonella* begins with the invasion of host intestinal epithelial cells and alteration of the host cellular mechanism (25). In addition, biofilms, the aggregation of bacterial cells surrounded by extracellular polysaccharide matrix, along with flagellar and fimbrial motility, function as critical virulence factors that facilitate the spread of infection between the hosts and their surrounding environment (26). *Salmonella* can adhere to various abiotic surfaces, such as water or feed buckets in horse stalls, where it forms biofilms that act as potential sources of contamination (7). The extracellular matrix of *Salmonella* biofilms increases the bacterial resistance to antibiotics, disinfectants, and the host immune system, making decontamination or treatment significantly more challenging (27, 28). On the other hand, swimming and swarming motility have been detected in *Salmonella* and reported as one of the major virulence factors (29). Motility enhances its ability to colonize, infect, and survive in diverse environments, contributing to the persistence of its pathogenicity (30).

Although most *Salmonella* infections are self-limiting, severe illness or systemic involvement necessitates antimicrobial therapy (31). The commonly used antibiotics for treating *Salmonella* infections in horses are ceftiofur, enrofloxacin, and gentamicin (32). However, the emergence of multidrug-resistant (MDR) *Salmonella* strains represents a primary factor contributing to the rising frequency of salmonellosis outbreaks (33–35). The resistance of *Salmonella* to these antibiotics in horses was reported in several studies (32, 36). For example, 10.2% of MDR *Salmonella* isolates with 20 different resistance patterns were observed among equines in the United States (32). Another study from the Netherlands reported 13% MDR *Salmonella* from horses where most of them were resistant to tetracycline (53%), ampicillin (34%), trimethoprim/sulfonamide (21%), and gentamicin (6%) (37). The precise time and manner in which MDR *Salmonella* emerged remain uncertain; however, the non-therapeutic use of antibiotics is considered a prime contributing factor (38). These resistant bacteria have the potential to cause subclinical or clinical infections in equines (39). However, these MDR *Salmonella* strains can pose a significant contamination risk, and their presence in necropsied horse samples may increase the potential for transmission. It is crucial to evaluate the risk factors and thoroughly characterize these *Salmonella* strains from necropsied horse samples. The aim of this study is to investigate the occurrence of *Salmonella* in clinical samples from horses, including a detection of serotype variations, biofilm formation, motility, antibiotic resistance pattern, and genotypic characterization of virulence and antimicrobial resistance (AMR) genes.

## MATERIALS AND METHODS

### Samples collection, *Salmonella* detection and confirmation, and serotyping

A total of 2,182 horses were submitted for necropsy to the Veterinary Diagnostic Laboratory at the University of Kentucky between January 2022 and December 2023. The majority of the horses exhibited symptoms related to the gastrointestinal tract, with some testing positive for *Salmonella* before necropsy. Samples were collected from different organs, including the intestine, lung, liver, kidney, colon, and feces of both hospitalized and non-hospitalized horses (Table S1). Following searing the surface of the organs with sterile blades, swab samples were obtained and plated on blood agar (BA) (Hardy Diagnostics, Santa Maria, CA, USA), hektoen (Hardy Diagnostics, Santa Maria, CA, USA), and eosin methylene blue (EMB) (Hardy Diagnostics, Santa Maria, CA, USA) plates. Fecal swabs were enriched in selenite broth, in addition to direct inoculation onto BA, hektoen, and EMB, and incubated at 37°C for 18–24 h. Enriched samples were further subcultured on hektoen and EMB agar plates and incubated at 37°C for 48 h. Black (in hektoen) and white (in EMB) colonies were selected for further studies as described before (40). Briefly, suspected colonies were selected, subcultured, and identified by matrix-assisted laser desorption–ionization time of flight mass spectrometry (MALDI-TOF) using the direct transfer method and a minimum score of 1.7 for genus identification using the Biotyper software (version 4.0; Bruker Scientific Corp., San Jose, CA, USA). Genomic DNA was extracted from the positive isolates using the boiling method as described before (41), and quality was checked using Thermo Scientific NanoDrop (Thermofisher, Lexington, KY, USA). Pure bacterial colonies were added to 100 µL distilled water, heated at 95°C for 10 min, and then cooled at 4°C for 10 min in the BioRad thermal cycler (Bio-Rad, Hercules, CA, USA). Centrifugation was performed at 14,000 rpm for 10 min. Then, 50 µL supernatants were collected and used as DNA template. To confirm the positive isolates, a PCR targeting the *invA* gene was performed using oligonucleotide primers listed in Table S2 (42). The amplification was performed in a 12.5 µL reaction volume. Each reaction consisted of 6.25 µL of 2× Green master mix (1 U *i-Taq* DNA polymerase, 2× PCR buffer, 3 mM MgCl_2_, and 0.4 mM dNTPs; Promega, Madison, WI, USA), 0.5 µL each of forward and reverse primers (10 µM), 1 µL DNA template, and 4.25 µL of PCR water. PCR was performed using the following conditions: initial denaturation at 95°C for 3 min, denaturation at 95°C for 30 sec, annealing at 60°C for 30 sec, extension at 72°C for 30 sec, repeated for 35 cycles, and a final extension at 72°C for 5 min. Serotyping was performed in the National Veterinary Services Laboratory (NVSL) following the Kauffmann–White–Le Minor Scheme (43). A conventional agglutination test employing anti-O and anti-H antisera was performed. The combination of O- and H-antigen results detected from the agglutination test was for the specific *Salmonella* serotype identification.

### Biofilm formation assay

Biofilm formation is one of the major virulence properties of *Salmonella*. To quantify the biofilm formation of isolated *Salmonella* spp., the experiment was conducted as described previously (44). Briefly, *Salmonella* isolates were inoculated in Luria–Bertani (LB) broth (BD Difco, Franklin Lakes, NJ, USA) and incubated at 37°C for 12 h. The bacterial culture was then diluted 1:100 in fresh LB medium and grown at logarithmic phase at 37°C for 6 h. A total of 100 µL of each diluted culture (OD_600_ = 0.05) was transferred to a 96-well plate. The plates were then incubated for 24 h at 37°C without shaking. After incubation, the plates were washed three times using 200 µL distilled water and allowed to dry. Subsequently, 125 µL of 0.01% crystal violet (CV) solution was added to all the wells containing the dried biofilms. The plates were then washed twice with sterile water and allowed to dry. The CV was dissolved using 30% acetic acid, and the absorbance was measured at a wavelength of 540 nm using Sunrise microplate reader (Tecan, Morrisville, NC, USA). Four replicate wells of each isolate were used. LB broth was used as a blank control (45).

The optical density (ODsample, ODs) could reflect the adhesion ability of biofilm, which was differentiated by the critical OD (ODcontrol, ODc). The ODc was calculated from the arithmetic mean of the absorbance of negative controls with the addition of three times the standard deviation (SD, ODc = mean absorbance of NC + 3(SD)). The following classification was applied for the categorization of biofilm formation level: no biofilm production (OD ≤ ODc), weak biofilm production (WBP; ODc ≤ OD ≤ 2 ODc), moderate biofilm production (MBP; 2ODc ≤ OD ≤ 4 ODc), and strong biofilm production (SBP; 4ODc ≤ OD) (45).

### Motility assay of *Salmonella* isolates

To assess the motility of our isolates, swarming and swimming motility assays were performed as described previously (46). The assays were conducted in 12-well plates (2.26 cm in diameter). Swarming motility was tested on plates containing Nutrient Broth (NB) (BD Difco, Franklin Lakes, NJ, USA), 0.5% glucose (w/v), and 0.5% bacteriological agar, while swimming motility was tested on plates with NB, 0.5% glucose, and 0.25% bacteriological agar (BD Difco, Franklin Lakes, NJ, USA). Using a sterile tip, an overnight *Salmonella* culture (OD_600_: 0.05) was lightly touched and gently spotted in the center of both swarming and swimming plates, followed by incubation at 37°C for 24 h. Turbid zone formation rates (in millimeters) were recorded at 5 h for swimming plates, and diameters of swarming *Salmonella* were measured at 12 h. *Salmonella* Typhimurium ATCC 14028 served as the positive control. Motility diameters were recorded in millimeters. Results included at least three independent assay observations.

### Molecular detection of virulence genes

To determine the virulence profile of the positive isolates, virulence genes were tested using PCR. DNA extraction was performed using the boiling method as described above (41). The targeted virulence genes of *Salmonella enterica* include structure and cell invasion genes (*invA* and *sopB*), regulatory protein genes (*hilA, hilC,* and *hilD*), fimbrial genes related to biofilm formation (*csgA* and *csgB*), effector protein genes (*sopB* and *avrA*), pathogenicity island genes (*spiA* and *spiC*), immune system evasion-related genes (*spvC*), oxidative stress survival-related genes (*sodC1*), magnesium homeostasis (*mgtC*), superoxide peroxidase-producing genes (*sodC1*), flagellar genes related to motility (*fliC, flhD, flgM, fliA,* and *motA*), and secretion system-related genes (*siiD* and *spvC*). Primers used in this study are listed in Table S2. PCR amplifications were performed in a final volume of 10 µL containing DNA template (1 µL), ×2 PCR Green Master mix (Promega, Madison, WI, USA) (5 µL), 10 pmol/µL of each primer (Thermofisher, Lexington, KY, USA) (0.5 µL), and 3 µL nuclease-free water. PCR amplifications using primers in this study were conducted at 94°C for 5 min, followed by 30 cycles of 94°C for 1 min; the corresponding temperatures for annealing (T_A_) are listed in Table S1 for 1 min, and 72°C for 1 min, and a final extension at 72°C for 5 min. All PCR amplifications were carried out in a BioRad thermal cycler (Bio-Rad, Hercules, CA, USA). The amplified DNA products were visualized using 1% (w/v) agarose gel stained with ethidium bromide and photographed using a gel documentation system (Bio-Rad, Hercules, CA, USA).

### Antibiotic susceptibility test (AST)

To assess the susceptibility profile of the positive isolates to antibiotics, the broth microdilution method was conducted using Thermo Scientific Sensititre Equine EQUIN2F (Thermofisher, Lexington, KY, USA) AST plate. Each plate includes 10 antimicrobial agents, such as amikacin (AK; 4–32 μg/mL), ampicillin (AMP; 0.25–32 μg/mL), ceftazidime (CAZ; 0.5–64 μg/mL), ceftiofur (XNL; 0.5–64 μg/mL), chloramphenicol (CL; 4–32 μg/mL), doxycycline (DOX; 2–16 μg/mL), imipenem (IMP; 1–8 μg/mL), tetracycline (TE; 2–8 μg/mL), trimethoprim/sulfamethoxazole (COT; 0.5/9.5-4/76 µg/mL), and gentamicin (GEN; 1–8 μg/mL) (Table S3). All isolates were grown on Columbia agar plates with 5% sheep blood (Becton Dickinson, NJ, USA) and incubated for 24 h at 37°C. The AST was performed according to the manufacturer’s instructions. Briefly, a 0.5 McFarland suspension (OD_600_ = 0.08–0.1) of the strain was prepared in distilled water, using a spectrophotometer, and then 10 µL of this suspension was mixed into the Sensititre Mueller Hinton broth. Each of the 96 wells was inoculated with 50 µL of the mixed suspension. The plate was incubated at 37°C, and the minimum inhibitory concentration (MICs) reading was taken after 18 h using the Thermo Scientific Sensititre OptiRead Automated Fluorometric Plate Reading System. The breakpoints for each antibiotic were determined according to the Clinical and Laboratory Standards Institute guidelines (CLSI 2024) (Table S3).

### Molecular detection of antibiotic resistance genes

To detect the antibiotic resistance genes among the isolates, PCR was conducted targeting specific genes. Genes that are responsible for the phenotypic aminoglycoside resistance (*aacA* [3]), beta-lactamase resistance (*bla_TEM_, bla_CTX-M_, bla_SHV2_,* and *bla_OXA-9_*), tetracycline resistance (*tetB*), amphenicol (*floR*), streptomycin resistance (*strA*), sulfonamide resistance (*sul2*), macrolides (*ermB2*), and quinolone resistance (*qnrB2*) were targeted for this genotypic resistance screening (Table S4). PCR amplifications were performed in a final volume of 10 µL containing DNA template (1 µL), ×2 PCR Green Mastermix (Promega, Madison, WI, USA) (5 µL), 10 pmol/µL of each primer (Thermofisher, Lexington, KY, USA) (0.5 µL), and 3 µL nuclease-free water. PCR amplifications using primers in this study were conducted at 94°C for 5 min, followed by 30 cycles of 94°C for 1 min; the corresponding temperatures for annealing (T_A_) are listed in Table S3 for 1 min, and 72°C for 1 min, and a final extension at 72°C for 5 min. All PCR amplifications were carried out in a BioRad thermal cycler (Bio-Rad, Hercules, CA, USA). The amplified DNA products were visualized using 1% (w/v) agarose gel stained with ethidium bromide and photographed using a gel documentation system (Bio-Rad, Hercules, CA, USA).

### Statistical analysis

Association between sample type and *Salmonella* prevalence was determined using Fisher’s Exact Test and descriptive statistics was performed to find out the 95% CI level. These analyses were performed in R using the stats package. A two-way analysis of variance (ANOVA) was conducted to assess the impact of two independent factors on the categorical outcome of biofilm formation and motility assays. The Complex Heatmap package in R was used to generate the hierarchical clustering heatmap (47). The correlation between AMR and virulence genes was calculated using Pearson’s correlation coefficient. The corrplot package was utilized to visualize the correlation matrix, providing an intuitive graphical representation of the correlations between variables (48). All the results were deemed significant if the *P*-value was less than 0.05.

## RESULTS

### Prevalence and serotyping of *Salmonella*

Out of 2,182 samples collected, *Salmonella* was recovered from 26 samples with an overall prevalence of 1.2%. These *Salmonella* were isolated from 26 individual horses. A significantly higher prevalence of 57.7% (15/26, 95% CI: 38.9%–74.5%, *P* < 0.0001) of *Salmonella* was observed in intestine samples of horses (Table 1). Significantly higher prevalence was observed in foals (61.5%) and female horses (76.9%) (Table 1). Additionally, 10 different *Salmonella* serotypes were detected within the positive isolates. The most prevalent serotype observed in this study was *Salmonella* Typhimurium (34.6% (9/26); 95% CI: 16.4%–52.8%), followed by *Salmonella* Thompson (7.7%; 2/26, 95% CI: 2.1%–24.2%). Other serotypes observed were *Salmonella* Hartford, *Salmonella* Anatum, *Salmonella* 4,(5), ,12:b:-, *Salmonella* 4,(5), 12:i:-, *Salmonella* Agbeni, *Salmonella* Bovismorbificans, *Salmonella* Enteritidis and *Salmonella* Mbandaka (Table 2)

**TABLE 1:** The distribution of *Salmonella* prevalence across various organs, age groups, and sexes of necropsied horses

Variables	Site isolated	No. of the positive isolates (Total = 26)	Prevalence (%)	Lower CI (%)	Upper CI (%)	p-value
Isolated site	Colon	7	26.92	13.70	46.08	<0.0001
	Feces	2	7.69	2.14	24.14	
	Kidney	1	3.85	0.68	18.89	
	Liver	1	3.85	0.68	18.89	
	Small intestine	15	57.69	38.95	74.46	
Age	Foal (0–1 year)	16	61.54	37.88	85.19	<0.05
	Yearling (>1–2 years)	2	7.69	4.73	10.64	
	Young (>2–4 years)	1	3.85	2.37	5.32	
	Adult (>4–15 years)	7	26.92	16.57	37.27	
						
Sex	Female	20	76.92	57.95	88.97	<0.05
	Male	6	23.08	11.03	42.05	

**TABLE 2:** Serotype prevalence of *Salmonella* spp

*Salmonella* serotypes	Isolate no. (Total= 26)	Prevalence (%)	Lower CI (%)	Upper CI (%)
4,(5), 12:b:-	2	7.69	2.14	24.14
4,(5), 12:i:-	2	7.69	2.14	24.14
Agbeni	1	3.85	0.68	18.89
Anatum	3	11.54	4.00	28.98
Bovismorbificans	1	3.85	0.68	18.89
Hartford	3	11.54	4.00	28.98
Enteritidis	1	3.85	0.68	18.89
Mbandaka	2	7.69	2.14	24.14
Thompson	2	7.69	2.14	24.14
Typhimurium	9	34.62	16.4	52.8

### Biofilm formation

**Fig 1:**
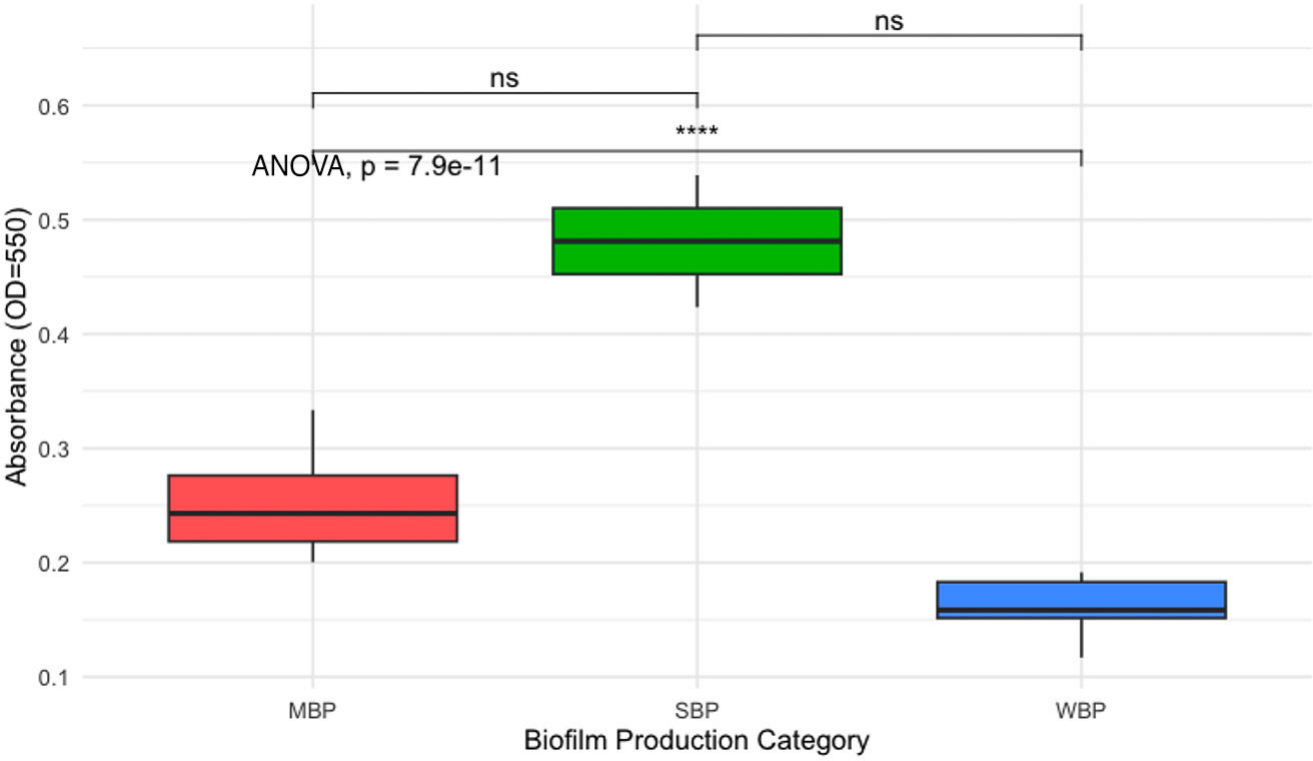
Biofilm production in *Salmonella* isolates. The figure shows a boxplot representing the absorbance (OD = 550) values across three categories of biofilm production in *Salmonella* isolates: moderate biofilm producers (MBP), strong biofilm producers (SBP), and weak biofilm producers (WBP). The absorbance values are indicative of the extent of biofilm formation, with higher absorbance reflecting stronger biofilm production. Results suggest that statistically significant differences are visible among all the categories of biofilm producers. SBP strains produces significantly higher amount of biofilm than MBP and WBP. *P*-value annotation legend: ns: non-significant; ****P < 0.0001.

To track the biofilm formation ability of the isolated *Salmonella* serotypes, all 26 isolates were subjected to CV assay. All the isolates were found to be biofilm producers, and among them, 7.7% (2/26, 95% CI: 2.1%–24.1%) were strong biofilm producers, 38.5% (10/26, 95% CI: 22.4%–57.5%) were moderate biofilm producers, and 53.8% (14/26, 95% CI: 35.4%–71.2%) were weak biofilm producers (Table 3; Table S5). Significant differences in biofilm production were observed among the isolates categorized as weak biofilm producers (WBP), moderate biofilm producers (MBP), and strong biofilm producers (SBP) (*P* < 0.01) (Fig. 1). All SBP isolates (E25 and E26) belonging to *Salmonella* Mbandaka serotype. Isolates belonging to *Salmonella* Thompson, *S*. Bovismorbificans, and *Salmonella* Enteritidis were MBP. In addition, 100% of *Salmonella* Agbeni and 77.8% of *Salmonella* Typhimurium serotypes were WBP (Table 4; Table S5).

**TABLE 3:** Prevalence of biofilm formation^*a*^

Category	No. of isolates	Prevalence (%)	Lower CI (%)	Upper CI (%)
MBP	10	38.46	22.43	57.47
SBP	2	7.69	2.14	24.14
WBP	14	53.85	35.46	71.24

^*a*^ MBP, moderate biofilm producers; SBP, strong biofilm producers; WBP, weak biofilm producers.

**TABLE 4:** Prevalence of biofilm formation in different *Salmonella* serotypes^*a*^

Serotype	MBP % (n= 9)	SBP % (n= 2)	WBP % (n=15)
4,(5), 12:b:-	50	0	50
4,(5), 12:i:-	50	0	50
Agbeni	0	0	100
Anatum	33.33	0	66.67
Bovismorbificans	100	0	0
Enteritidiis	100	0	0
Hartford	66.67	0	33.33
Mbandaka	0	100	0
Thompson	100	0	0
Typhimurium	22.22	0	77.78

^*a*^ MBP, moderate biofilm producers; SBP, strong biofilm producers; WBP, weak biofilm producers.

### Motility determination of *Salmonella* isolates

The isolates were tested for their ability to move across both swarming and swimming plates. All isolates exhibited swarming behavior, forming smooth, featureless colonies, with motility measurements averaging more than 2.6 ± 0.5 mm. The highest swarming motility was recorded in isolates belonging to *Salmonella* Typhimurium (E14) and *Salmonella* Hartford (E21), with 3.5 ± 0.5 and 3.6 ± 0.5 mm diameters, respectively (Table S6; Fig. S1). Additionally, swimming motility was observed across all the isolates with an average of 4.0 ± 0.9 mm diameter. The highest swimming motility was observed in isolates belonging to *Salmonella* Typhimurium (E12 and E17) with a 6 mm diameter each (Table S5; Fig. S1B).

### Virulence genes detection within *Salmonella* isolates

Overall, 26 virulence genes were screened among the *Salmonella* isolates. Our results showed that 100% of the isolates contained *invA, hilA, mgtC, spiA, filA, motA, figG, figH, figC, fimC, fimD, fimF, fimH csgA,* and *csgB* genes. These genes are responsible for several virulence factors, including invasion, intracellular survival, motility, biofilm formation, and regulatory genes (Table 5). Additionally, the type III secretion system (T3SS) effector protein genes, such as *avrA* and *siiD* possessed the second highest prevalence (96.1%) among the isolates. Other gene predispositions included flagellar protein gene *fliC* and superoxide dismutase producing gene *sodC1* (46.2%), regulatory protein gene *hilC* and invasion gene *sipD* (88.5%), and pathogenicity island gene *spiC* (73.1%) were also detected. Only 30.8% of *spvC* genes were detected (Table 5; Fig. S2).

**TABLE 5:** Prevalence of virulence genes within the isolated bacteria

Group	Function	Gene	Number of the positive isolates	Prevalence %
Invasion related gene	T3SS component	*invA*	26	100
	T3SS component	*sipD*	23	88.5
	T3SS component	*spiC*	19	73.1
	T3SS component	*spiA*	26	100
Immunity	Invade host immunity	*avrA*	25	96.2
	Invade host immunity	*sopB*	23	88.5
	Suppress host immune response	*spvC*	8	30.8
	Defense against oxidative stress	*sodC1*	12	46.2
Survival	Survival inside macrophage	*mgtC*	26	100
	Colonization inside host	*siiD*	25	96.2
Motility	Flagellar component	*fliC*	12	46.2
	Flagellar component	*figC*	26	100
	Flagellar component	*figH*	26	100
	Flagellar component	*filA*	26	100
	Fimbrial assembly chaperone	*fimC*	26	100
	Type one fimbriae usher	*fimD*	26	100
	Structural fimbrial subunit	*fimF*	26	100
	Fimbrial tip adhesin	*fimH*	26	100
	Flagellar motor component	*motA*	26	100
Biofilm	Biofilm formation	*csgA*	26	100
	Biofilm Formation	*csgB*	26	100
Regulatory gene	Transcriptional regulation of SPI-1	*hilA*	26	100
	Transcriptional regulation of SPI-1	*hilC*	17	65.4
	Transcriptional regulation of SPI-1	*hilD*	17	65.4

### Antimicrobial susceptibility test

**Fig 2:**
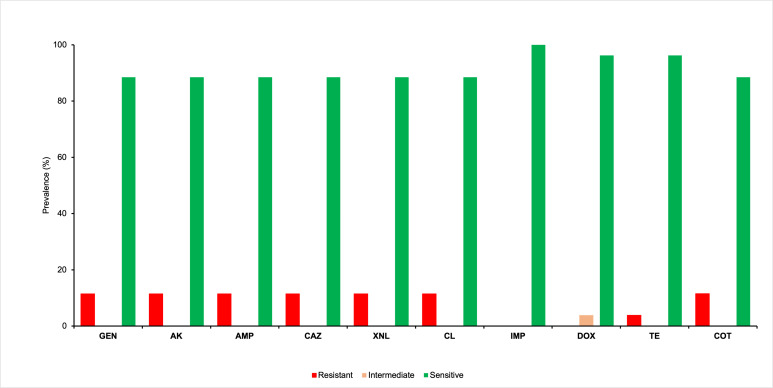
Antibiotic resistance profiles of *Salmonella* enterica isolates. Amikacin (AK), ampicillin (AMP), ceftazidime (CAZ), ceftiofur (XNL), chloramphenicol (CL), doxycycline (DOX), imipenem (IMP), tetracycline (TE), trimethoprim/sulfamethoxazole (COT), and gentamicin (GEN). Approximately 11.5% of the isolates were resistant to XNL, GEN, COT, CL, CAZ, AMP, and AK, while 3.9% of the isolates showed resistance to TE.

Antimicrobial susceptibility test was performed using broth microdilution for 26 *Salmonella* isolates. Three isolates (11.5%) were found resistant to aminoglycosides (gentamicin and amikacin), penicillin (ampicillin), cephalosporins (ceftazidime and ceftiofur), chloramphenicol, and sulfonamides (trimethoprim/sulfamethoxazole). Besides, 3.9% of the isolates possessed resistance to tetracycline. Imipenem (100%) was found to be the most effective against these isolates, followed by doxycycline (96.2%) (Table S7; Fig. 2). Among the 26 isolates, three (11.5%) exhibited MDR. The MDR isolates exhibited two distinct resistance patterns, showing resistance to five and six antibiotic groups, as presented in Table 6. Multidrug resistant *Salmonella* serotypes were *Salmonella* Typhimurium and *Salmonella* Mbandaka. Both *S*. Mbandaka (2/2, 100%) detected in this study exhibited resistance to the same group of antibiotics, including amikacin, ampicillin, ceftazidime, ceftiofur, chloramphenicol, gentamicin, and trimethoprim/sulfamethoxazole. In contrast, 33.4% (1/3) *S*. Typhimurium showed resistance to tetracycline in addition to the aforementioned antibiotics (Table 6).

**TABLE 6:** Multidrug resistance profile of the *Salmonella* isolates^*a*^

MDR phenotypes	No. of antibiotics group	Name of the groups	No. of isolates (%)	Serotypes	MDR prevalence
AK, AMP, CAZ, XNL, CL, GEN, COT	5	Aminoglycosides, penicillins, cephalosporins, phenicol, sulfonamides	2 (7.69)	*S*. Mbandaka	11.53%
AK, AMP, CAZ, XNL, CL, GEN, TE, COT	6	Aminoglycosides, penicillins, cephalosporins, phenicol, tetracyclines, sulfonamides	1 (3.85)	*S*. Typhimurium	

^*a*^ AK, amikacin; AMP, ampicillin; CAZ, ceftazidime; XNL, ceftiofur; CL, chloramphenicol; DOX, doxycycline; GEN, gentamicin; IMP, imipenem; TE, tetracycline; COT, trimethoprim/sulfamethoxazole.

### Prevalence of antibiotic resistance genes

**Fig 3:**
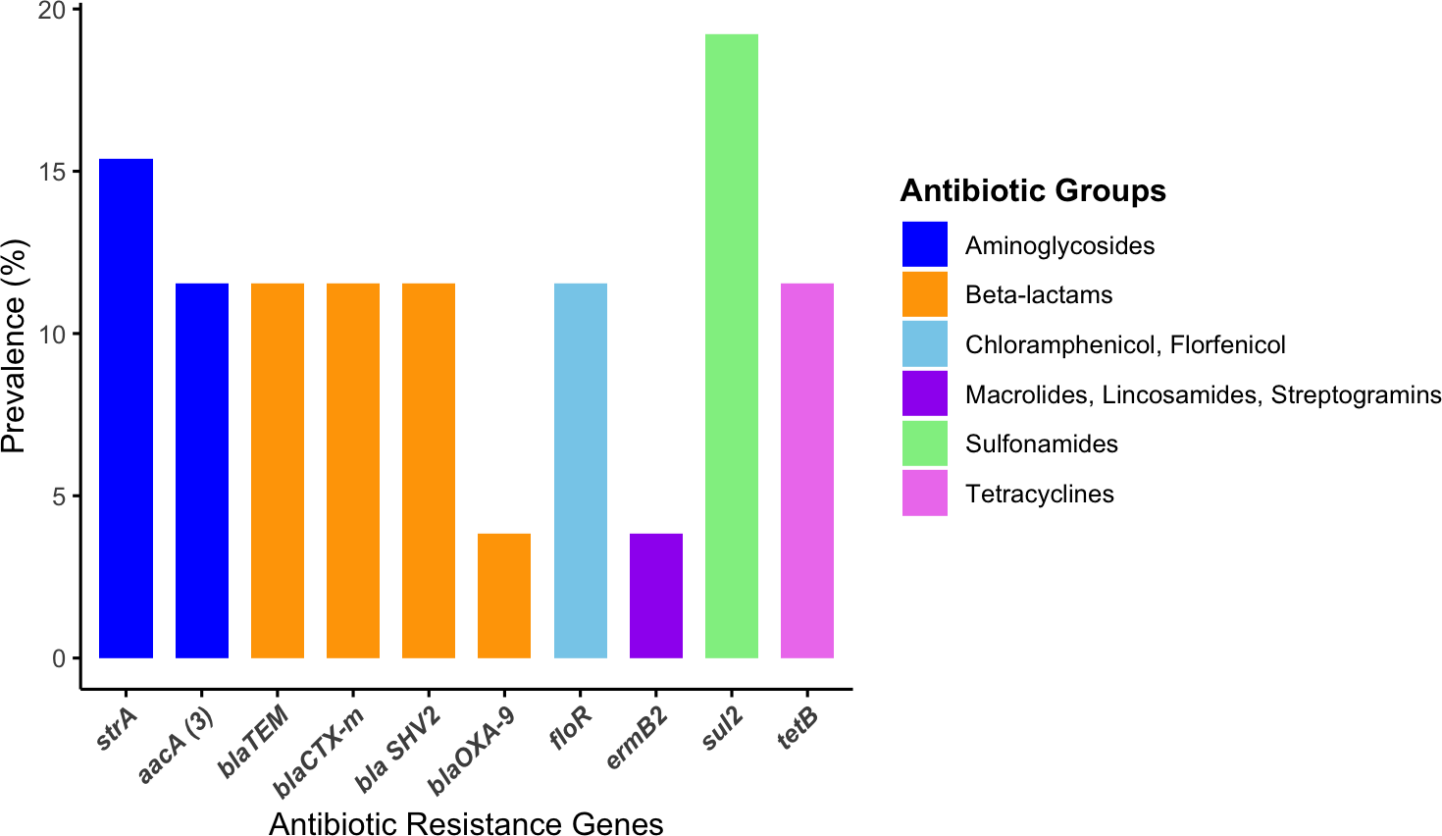
Prevalence of antimicrobial resistance (AMR) genes by function category. Our results showed that sulfonamide-resistance gene (*sul2*) was 19.2% prevalent among the isolates.

Antibiotic resistance genes were detected by PCR among these *Salmonella* isolates. It was observed that the sulfonamide-resistance gene (*sul2*) was identified as the most prevalent (19.2%) gene among the isolates. (Fig. 3). Beta-lactamase-producing genes, including *bla_TEM_, bla_CTX-M_,* and *bla_SHV-2_*, were identified in 11.5% of the isolates, while 3.8% of the isolates contain *bla_OXA-9_* genes. The aminoglycoside-resistance gene (*aacA*) was identified in 11.5% of the isolates, while the amphenicol resistance gene (*floR*) and tetracycline resistance gene (*tetB*) were detected in 11.5% of the isolates. Furthermore, it was observed that 15.4% of the isolates contained streptomycin resistance gene (*strA*), while only 3.8% contained the deduced macrolide (*ermB2*) and quinolone (*qnrB2*) resistance genes. The prevalence of all antibiotic resistant genes are shown in Table S8.

### Correlation between phenotypic and genotypic resistance of *Salmonella* isolates

**Fig 4:**
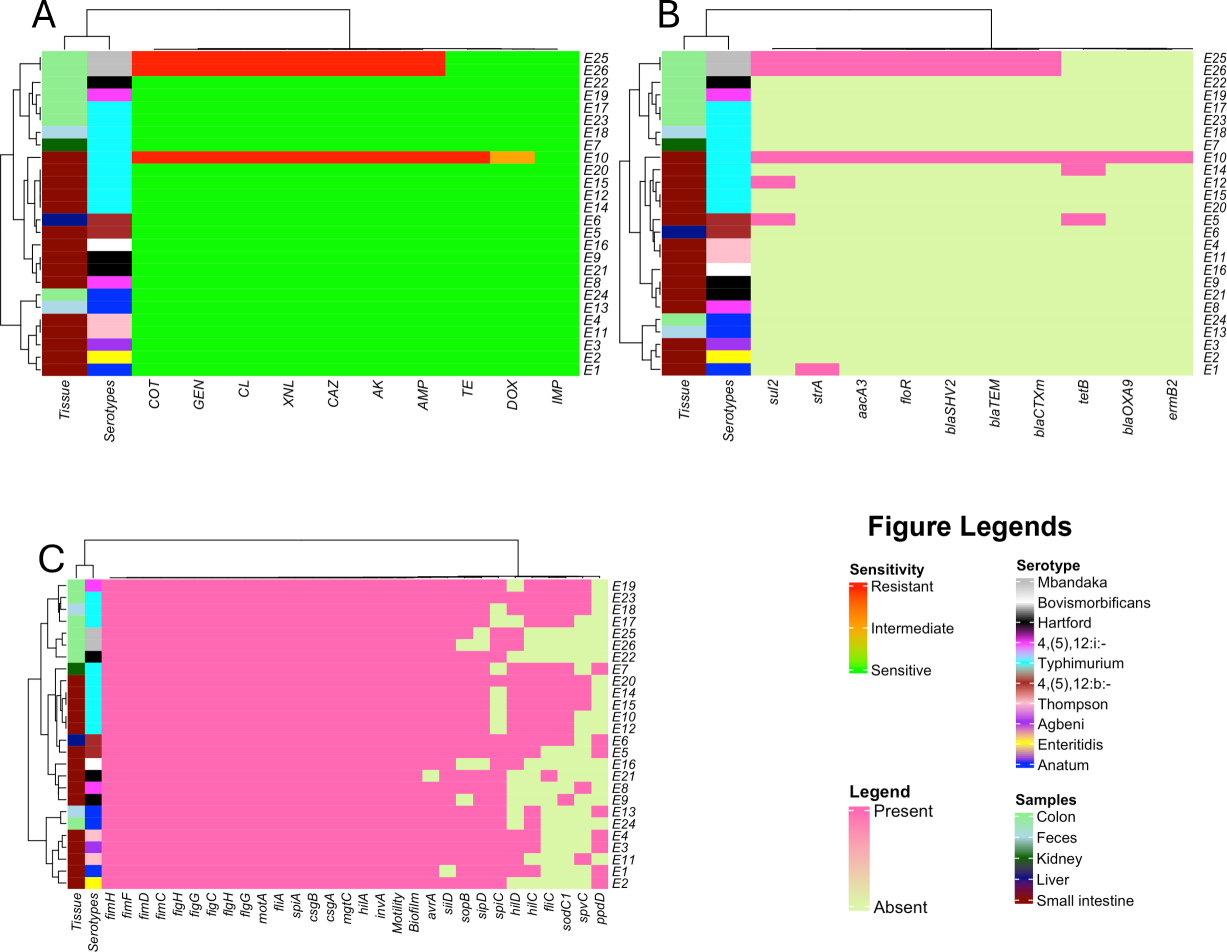
Heatmaps showing phenotypic and genotypic resistance profiles with clustering as a dendrogram. The clustering of isolates is based on their (A) phenotypic resistance pattern. Amikacin (AK), ampicillin (AMP), ceftazidime (CAZ), ceftiofur (XNL), chloramphenicol (CL), doxycycline (DOX), imipenem (IMP), tetracycline (TE), trimethoprim/sulfamethoxazole (COT), gentamicin (GEN). (**B**) Presence and absence of AMR genes. (C) presence and absence of virulence genes.

Hierarchical clustering heatmaps were generated to identify patterns and group similarities among the isolates based on their isolation site, serotype, phenotypic and genotypic AMR characteristics, as well as their virulence. The isolates, organized by serotype and isolation site, demonstrated distinct clustering in Fig. 4. A higher prevalence of *S*. Typhimurium was observed across various sample types, including the small intestine, kidney, feces, and colon. *S*. Anatum was isolated from different locations including the small intestine, feces, and colon, while *Salmonella* Mbandaka exclusively recovered from colon tissue. In contrast, *S*. Hartford and *S*. Enteritidis were solely isolated from the small intestine (Fig. 4).

The heatmap generated from the phenotypic AMR profiles revealed that isolates E25 and E26 isolated from the colon, which belonged to *Salmonella* Mbandaka serotype clustered together, exhibiting resistance to AK, AMP, CL, CAZ, XNL, GEN, and COT. Additionally, MDR *Salmonella* Typhimurium isolate (E10) clustered with other *Salmonella* Typhimurium isolates recovered from the small intestine that showed resistance to AK, AMP, CL, CAZ, XNL, GEN, TE, and COT. In contrast, rest of the isolates displayed complete sensitivity to all the antibiotics tested in this study (Fig. 4A).

A similar clustering pattern was observed for isolates E10, E25, and E26 in the heatmap based on their AMR genes, where these isolates contained *sul2, strA, aacA (3), floR, bla_SHV-2_, bla_TEM_,* and *bla_CTX-m_* genes (Fig. 4B). Additionally, E14 and E5 have the *tetB* gene, and the *sul2* gene was found to be common between isolates E5 and E12.

The heatmap generated based on the virulence profiles revealed different clustering than AMR heatmap (Fig. 4C). The majority of the virulence genes were present among the isolates. Clustering of isolates E17, E18, E19, and E23 was observed in first clade, where E17, E18, and E23 belonged to *Salmonella* Typhimurium and E19 belonged to *Salmonella* 4,(5), 12:i:- serotype. Isolate E23 had all the virulence genes present, except *ppdD*, and E19 lacked *hilD* and *ppdD*. Interestingly, isolates E25 and E26 also clustered together; however, the *sopB* gene responsible for invading host cell immunity was present in E25 but absent in E26 (Fig. 4C).

**Fig 5:**
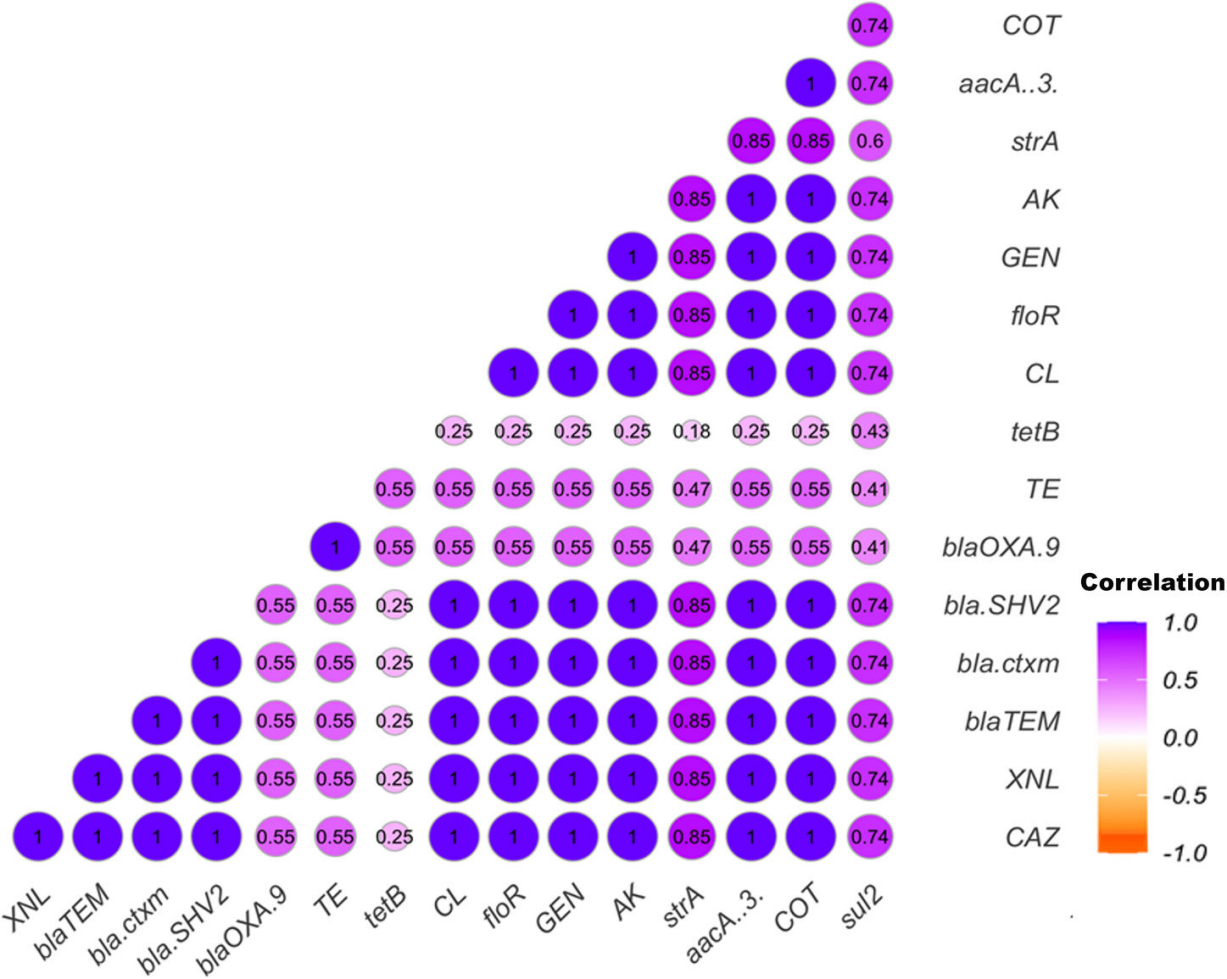
Pearson’s correlation coefficient indicating the relationship between phenotypic and genotypic antibiotic resistance, with associated significance levels. Correlation matrix shows correlation between phenotypic antimicrobial resistance and antimicrobial resistance genes. Amikacin (AK), ampicillin (AMP), ceftazidime (CAZ), ceftiofur (XNL), chloramphenicol (CL), doxycycline (DOX), imipenem (IMP), tetracycline (TE), trimethoprim/sulfamethoxazole (COT), gentamicin (GEN).

Pearson’s correlation coefficient analysis was conducted to assess the correlation between phenotypic and genotypic antimicrobial resistance, along with the significance levels. All antibiotics showed significant positive correlations (*P* ≤ 0.05) with each other (Fig. 5). Additionally, the correlation matrix of AMR genes revealed a strong positive relationship between *floR* and *aacA* (3) (r = 1; *P* < 0.001) and among the beta-lactamase-producing genes *bla_TEM_, bla_SHV-2_, and bla_CTX-m_* (r = 1; *P* < 0.001). A similar trend was observed between the *aacA* (3) gene and the beta-lactamase-producing genes, except for *bla_OXA-9_*. Further details are provided in Fig. 5.

## DISCUSSION

The equine industry has a significant economic impact on the United States, contributing approximately $177 billion per year (49). The equine sector is associated with an estimated 2.2 million jobs, counting both direct and indirect employment (49). The presence of *Salmonella* in horses can pose a risk to both the equine farms and their associated personnel, as there is a high potential for *Salmonella* infections to be transferred between different hosts. Therefore, it is crucial to conduct monitoring to detect the presence of *Salmonella* to prevent its epidemic in both animals and humans. Our study investigates the presence of various *Salmonella* serotypes in clinical samples submitted to a veterinary diagnostic laboratory. The study also explored their antimicrobial resistance profiles and virulence factors, including biofilm formation, motility, as well as AMR, and virulence associated genes.

In this study, *Salmonella* was identified in samples collected from necropsied horses, with a prevalence of 1.2%. This prevalence is lower than previous findings obtained from fecal laboratory testing conducted as part of the National Animal Health Monitoring System (NAHMS) Equine 2015–16 in the USA (2.0%) and from hospitalized horse feces in Chile (3.85%) (11, 50). However, some studies reported higher *Salmonella* shedding in colic-affected horses (51). One of the studies in South Africa showed that the prevalence of *Salmonella* in necropsied horses with a history of colic was 59% (52). The presence of *Salmonella* infections in clinical samples of necropsied horses is a public health and biosurity concern. Fecal shedding of *Salmonella* occurred in hospitalized horses posing a potential risk of transmission to humans, especially the horse owners and farmers, as well as other hospitalized animals. Previous studies reported that diseased animals are more susceptible to fecal shedding of *Salmonella* than healthy animals (5).

Our study has found a higher prevalence of *Salmonella* in the intestinal samples (57.7%). The intestinal samples had a higher prevalence because the bacteria invade the intestinal epithelial cells by inducing membrane ruffling in intestinal cells, which causes them to intake the bacteria (53). Although the cecum is recognized as a major predilection site for *Salmonella*, this study did not include cecum samples. This was due to the sampling process being conducted through the diagnostic laboratory, where testing was performed at the discretion of veterinarian or pathologists, which did not include cecal samples. The most common serotype reported in horses was *Salmonella* Typhimurium; however, other serotypes including *Salmonella* Enteritidis, *Salmonella* Abortusequi, *Salmonella* Heidelberg, *Salmonella* Newport, *Salmonella* Agona, *Salmonella* Anatum, and other “atypical” serotypes were also found (32, 54). In this study, *Salmonella* Typhimurium was the most prevalent, and this result aligns with the previous studies (11, 37). *Salmonella* Typhimurium is the predominant serotype responsible for foodborne illnesses globally. Its increased prevalence in horses poses a significant public health risk within the equine industry. However, other serotypes are equally important reported in horses previously (5, 12–14).

Biofilms are complex microbial communities that attach to surfaces and are embedded in a self-produced extracellular matrix. The extracellular matrix impedes the activity of antibiotics by preventing their penetration into bacterial cells, thus preventing the antibiotics from reaching their intended target receptors (55). Our results showed that all the isolates were biofilm producers, whereas 7.7% were strong biofilm producers. *Salmonella* Typhimurium and *Salmonella* Thompson serotypes were among moderate biofilm producers and *Salmonella* Mbandaka were among strong biofilm producers. Our findings were in accordance with previous studies (56, 57). The cell surface attachment and biofilm formation of *Salmonella* are mediated by amyloid-like cell-surface proteins that are highly aggregative and non-branching, and encoded by the Curli protein (csg) operon (58). Our results revealed that all our isolates contain the *csgA* and *csgB* genes, which align with the observed phenotypic outcome of biofilm formation (Fig. 5C). In addition to biofilm, all the isolates found motile where they showed both swimming and swarming motility. Strong swarming and swimming motility were observed among 19.2% and 30.8% isolates, respectively. *Salmonella* Typhimurium was found highly motile in both types of motility (swimming and swarming), which has been reported in the previous studies (59). All the isolates in this study were found to carry the flagellar and fimbrial genes, including *fliA, motA, flgG, flgH, fimC, fimD, fimF, fimH, figC*, and *figG*. The presence of such genes can be responsible for their motility as described previously (30, 60). This result aligns with the phenotypic result of the motility test where all the isolates were found to be motile (Fig. 5C).

The ability of *Salmonella* to invade host cells is a pivotal stage in its pathogenesis, facilitating bacterial survival, spread, and infectivity (61). The presence of genes associated with invasion and intracellular survival enables *Salmonella* to efficiently invade the host’s intestinal epithelial cells and persist within *Salmonella*-containing vacuoles. These genes also play a role in modulating the immune system, contributing to the pathogen’s virulence (27). The *Salmonella* pathogenicity island encodes the majority of these virulence-related genes, which are also exceptionally conserved across serotypes (62). *Salmonella* pathogenicity island 1 (SPI-1) contains the invasion-related genes *invA* and *hilA*, from which *hilA* is a key regulator for *invA* gene (63). These genes were identified in all the isolates in this study. They have been also reported in a similar study in samples collected from poultry. Genes related to type III secretion system (T3SS) formation were also detected including *spiA* (100%) and *sipD* (88.5%), as reported in previous studies (64, 65). Additionally, *Salmonella* plasmid virulence (*spv*) genes are plasmid-dependent and are serotype-specific in *Salmonella*. Our study detected *spvC* gene among 30.8% of the isolates, and the highest prevalence was observed among *Salmonella* Typhimurium serotypes and has also been reported in *Salmonella* from poultry in a previous study (66).

Though *Salmonellosis* causes self-limiting gastroenteritis in humans, antimicrobial treatments are necessary for the complete removal of this bacteria. However, AMR is developing in an upward manner, and AMR in *Salmonella* has become a threat to public and animal health. The prevalence of MDR *Salmonella* isolates is steadily increasing in horses. Between 2001 and 2013, *Salmonella* isolated from hospitalized horses at Cornell University showed resistance to multiple antibiotics, including amoxicillin–clavulanic acid (29%), ampicillin (45.5%), cefazolin (42.2%), cefoxitin (27.5%), ceftiofur (37.3%), chloramphenicol (45.2%), and tetracycline (46.1%). In this study, 11.5% of isolates were MDR, and the AMR pattern includes gentamicin (11.5%), amikacin (11.5%), ampicillin (11.5%), ceftazidime (11.5%), ceftiofur, chloramphenicol (11.5%), tetracycline (3.9%), and trimethoprim/sulfamethoxazole (11.5%). Similarly, a previous study has been reported MDR prevalence (10.2%) within the *Salmonella* isolates from horses (32). In comparison, another study reported a higher prevalence of MDR *Salmonella* (57%) in horses (36). The wide range of sampling locations across the USA along with large number of samples, and the extended 13-year duration of the study may have contributed to the variation in these results. Additionally, they evaluated different antimicrobial regimens, which is an additional cause of MDR strains with a high affinity (41). However, our study found imipenem to be the most efficacious drug against *Salmonella*, consistent with the previous studies conducted on equines in the USA (36). A possible explanation is that imipenem is less common in veterinary practice and restricted to human use only (67).

To confirm the agreement between phenotypic and genotypic antimicrobial resistance of the isolates, PCR detection of AMR genes was performed. Beta-lactamase-producing genes *bla_TEM_, bla_CTXM_,* and *bla_SHV2_* were detected in three isolates (11.5%), and these isolates were resistant to extended-spectrum cephalosporins. Multiple beta-lactamase-producing genes were reported previously in several studies in *Salmonella* isolated from horses (68, 69). Our study identified a *Salmonella* isolate carrying the *bla_OXA-9_* gene, which is also associated with cephalosporins resistance. Tetracycline resistance gene *tetB* was detected among three isolates; however, only one isolate was resistant to tetracycline. Because of their evolutionary limit, the other two might downregulate the *tetB* gene expression (70). The study highlights the critical role of monitoring *Salmonella* in horses, given its potential impact on public health, biosecurity, and the equine industry. Despite the relatively low prevalence of *Salmonella* in clinical samples compared with previous studies, the detection of various virulence factors and AMR genes, including those associated with biofilm formation and motility, raises concerns about the persistence and spread of these pathogens. These findings highlight the challenges in managing *Salmonella* infections within equine populations, emphasizing the critical need for continuous, comprehensive surveillance to minimize transmission risks to humans, other animals, and the environment.

### Conclusion

Our study highlights the pressing concern of AMR in *S. enterica* isolates from horses, along with significant virulence potential and biofilm formation capability. The presence of MDR and virulent *Salmonella* serotypes in horses poses a potential threat to the equine industry. *Salmonella* Typhimurium was the predominant serotype detected in this study, alongside nine other serotypes. Swarming and swimming motility were observed in all isolates, along with biofilm production. Three isolates were identified as MDR with the presence of AMR genes. A significant number of isolates also harbored virulence genes. The detection of such isolates demonstrates the threat of pathogenic *Salmonella* outbreaks in horses, as well as the potential risk to human health, especially the horse owners and farmers. Fecal shedding of *Salmonella* can lead to nosocomial transmission, and spreading the infection to uninfected horses within hospital settings. The characterization of these isolates provides an accurate profile of *Salmonella*, highlighting the emerging threat posed by these pathogens. These findings underscore the need for comprehensive surveillance and innovative approaches to manage and mitigate the impact of these pathogens on equine health and beyond. Comprehensive analysis using whole genome sequencing is essential to fully characterize the AMR and virulence gene profiles. Furthermore, studies on transmission dynamics employing whole genome sequencing are crucial to identify the source of *Salmonella* infections in equine populations and to understand its potential dissemination to the environment. These studies will assist hospitals and other authorities in identifying the source of contamination and implementing necessary biosecurity measures to prevent *Salmonella* transmission.

## SUPPLEMENTAL MATERIAL

Figure S1 spectrum.02501-24-s0001.tif Swarming and swimming motility of *Salmonella* isolates.

Figure S2 spectrum.02501-24-s0002.png Prevalence of virulence genes by their function category.

Supplemental figures legends spectrum.02501-24-s0003.docx Legends for supplemental figures.

Supplemental tables spectrum.02501-24-s0004.docx Tables S1 to S8.

ASM does not own the copyrights to Supplemental Material that may be linked to, or accessed through, an article. The authors have granted ASM a non-exclusive, world-wide license to publish the Supplemental Material files. Please contact the corresponding author directly for reuse.
