# Supplementary figures and images for: Phenotypic and genotypic characterization of antimicrobial resistance and virulence profiles of Salmonella enterica serotypes isolated from necropsied horses in Kentucky
Source: Microbiol Spectr. 2025 Jan 23;13(3):e02501-24. doi: 10.1128/spectrum.02501-24 (PMC11878045; doi:10.1128/spectrum.02501-24)

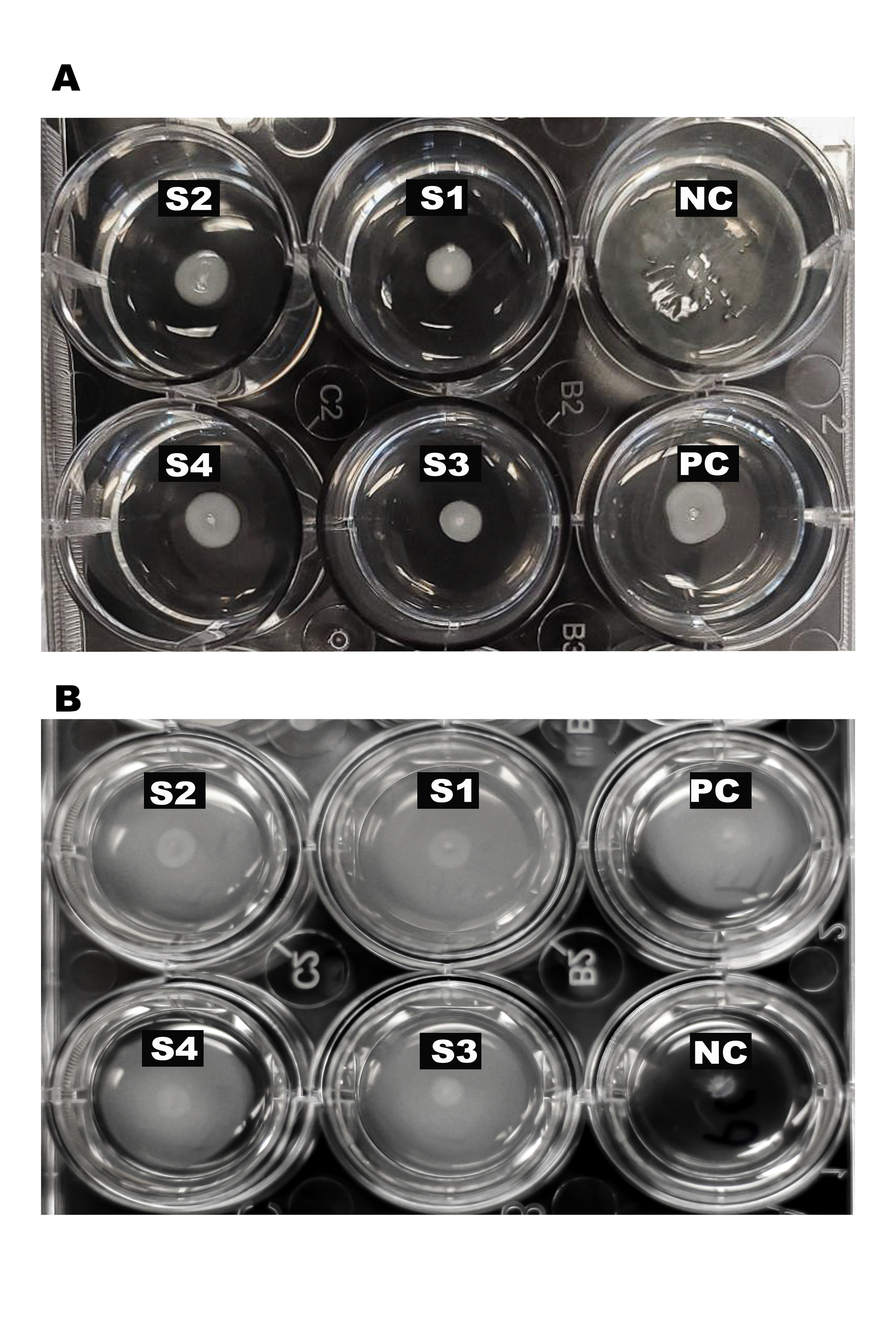

Supplement: Figure S1 — Swarming and swimming motility of Salmonella isolates. [file spectrum.02501-24-s0001.tif]

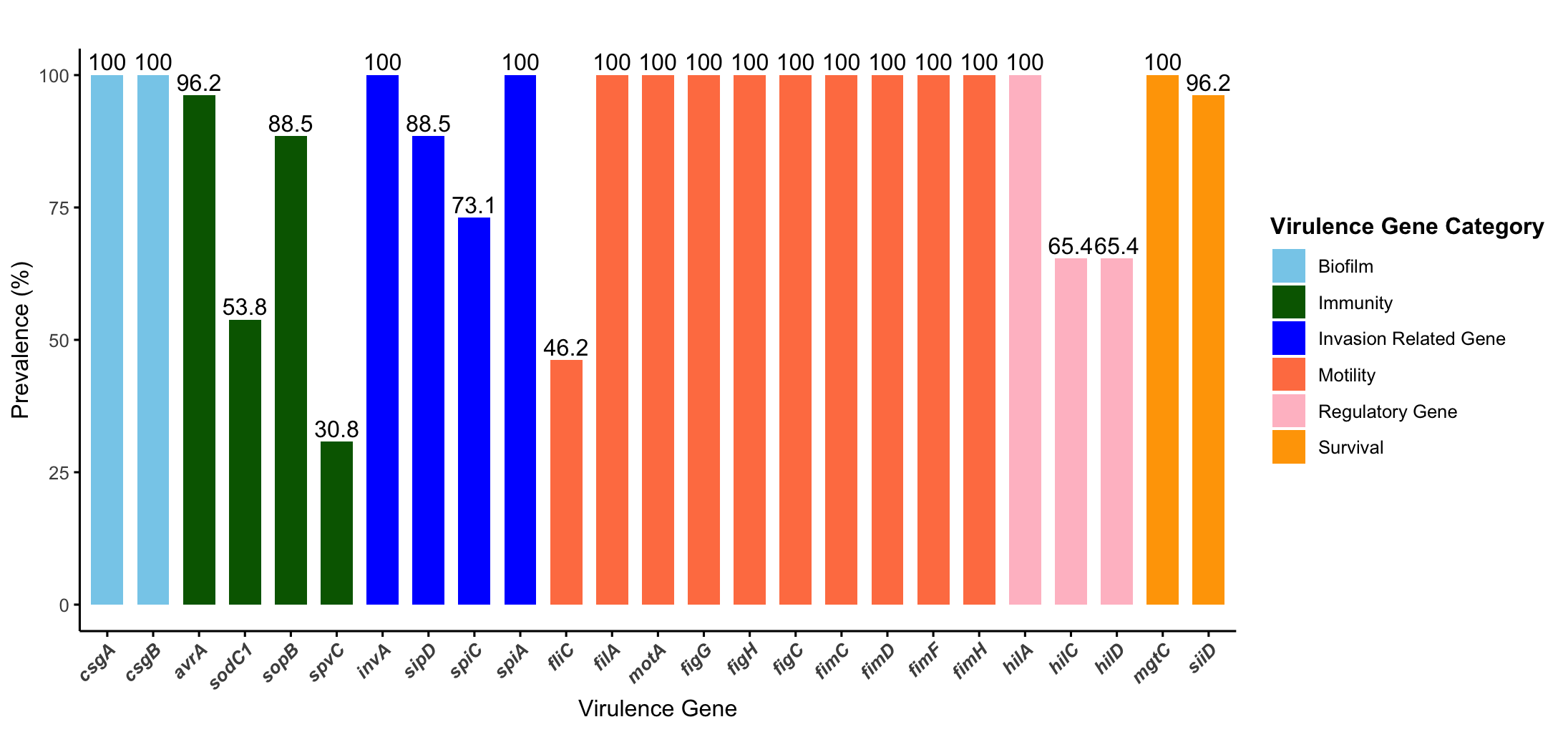

Supplement: Figure S2 — Prevalence of virulence genes by their function category. [file spectrum.02501-24-s0002.png]
