# Supplementary material for: Phenotypic and genotypic characterization of antimicrobial resistance and virulence profiles of Salmonella enterica serotypes isolated from necropsied horses in Kentucky
Source: Microbiol Spectr. 2025 Jan 23;13(3):e02501-24. doi: 10.1128/spectrum.02501-24 (PMC11878045; doi:10.1128/spectrum.02501-24)
Supplement: Supplemental figures legends — Legends for supplemental figures. [file spectrum.02501-24-s0003.docx]

**Supplementary Figures Legends**

**Supplementary Figure 1.** Swarming and Swimming motility of *Salmonella* isolates. A. Swarming motility of *Salmonella* in nutrient broth (0.5% agar) supplemented with glucose. and B. Swimming motility of *Salmonella* in nutrient broth (.25% agar) supplemented with glucose. Here, PC: Positive Control, NC: Negative Control, S1-S4: Sample 1-4.

**Supplementary Figure 2.** Prevalence of Virulence Genes by their Function Category. Our results showed that isolates were 100% prevalent for Biofilm (*csgA* and *csgB*), Motility (*figC, figG, figH, filA, fimC, fimD, fimH,* and *motA*), regulatory genes (*hilA*) and invasion-related genes (*invA* and *spiA*).
