# Supplementary material for: Phenotypic and genotypic characterization of antimicrobial resistance and virulence profiles of Salmonella enterica serotypes isolated from necropsied horses in Kentucky
Source: Microbiol Spectr. 2025 Jan 23;13(3):e02501-24. doi: 10.1128/spectrum.02501-24 (PMC11878045; doi:10.1128/spectrum.02501-24)
Supplement: Supplemental tables — Tables S1 to S8. [file spectrum.02501-24-s0004.docx]

**Supplementary Table 1.** History and pathological diagnosis of necropsied horses.

| **Strain** | **Hospitalization** | **History** | **Pathological Diagnosis** |
| --- | --- | --- | --- |
| E1 | Yes | Presented to hospital for colic | Large colon impaction with marked gas distention |
| E2 | Yes | Presented to the hospital with chronic colic. | Moderate lymphoplasmacytic and eosinophilic colitis with submucosal edema |
| E3 | No | Colic surgery-colon torsion | Gastric rupture with peritonitis |
| E4 | Yes | Presented with severely painful surgery found large colon volvulus | Large colon: multifocal, severe transmural hemorrhage and suppurative colitis; Brainstem: severe nonsuppurative and eosinophilic encephalitis, EPM positive |
| E5 | No | Scours last night, normal temperature; found dead next day. | Gastric ulceration, rupture, and peritonitis; Enterocolitis. |
| E6 | No | Diarrhea for two weeks | Necrotizing enterotyphlocolitis, *Salmonella* sp. and *Clostridium difficile* isolated; Necrotizing hepatitis; Pulmonary pyogranuloma |
| E7 | Yes | Found dead on the field. | Acute necrotizing hepatitis (suspect Tyzzer's Disease); Chronic urachitis, umbilical arteritis (*Streptococcus zooepidemicus*). |
| E8 | Yes | Presented with respiratory distress; *R. equi* diagnosed by tracheal culture | Bronchopneumonia, R. equi; Acute interstitial pneumonia |
| E9 | No | Sudden death | Necrotizing and suppurative colitis. |
| E10 | Yes | Presented with abdominal discomfort; pyloric stricture | Pyloric stricture |
| E11 | Yes | Diarrhea; septic right hind. | Enteric Salmonellosis; Septic arthritis and tenosynovitis, right hind fetlock and flexor tendons |
| E12 | No | Found dead | Mesothelioma with metastasis to lung |
| E13 | No | Diarrhea, positive for *Salmonella* | Enteric salmonellosis |
| E14 | Yes | Febrile episodes over a weekend | Actinobacillus septicemia; Pulmonary and renal thrombosis; Valvular endocarditis |
| E15 | No | Increased temperature, found dead the next day | Necro-hemorrhagic pleuropneumonia; Large colon displacement |
| E16 | Yes | Diarrhea, increased respiratory rate | Neutrophilic colitis and pelvic flexure necrosis, *Salmonella* culture positive |
| E17 | Yes | Surgery due to the colic-large colon volvulus | Acute suppurative enteritis; Acute colonic necrosis and hemorrhage |
| E18 | No | Fever, weight loss, diarrhea | Enteric salmonellosis |
| E19 | Yes | Diarrhea, positive for *Salmonella*, lameness-humanely euthanized | Left front, right front, right hind: suppurative arthritis, synovitis and tendinitis; Intestine: neutrophilic colitis |
| E20 | No | Found dead | Small intestinal rupture with peritonitis; Moderate number of ascarids |
| E21 | Yes | Presented with acute signs of hypotensive shock-perforated GI tract | Stomach: Severe necro ulcerative gastritis with perforation and peritonitis; Duodenum: Neutrophilic enteritis; Rotavirus A positive |
| E22 | Yes | Septic peritonitis, GI rupture | Large colon rupture; Chronic peritonitis; Intestinal parasitism |
| E23 | Yes | Presented with colic pain, extensive adhesion in the surgical exploration, humanely euthanized | Chronic lymphadenitis, R. equi; Acute to subacute enteritis; Acute segmental intestinal hemorrhage (compatible with volvulus). |
| E24 | Yes | Ongoing pneumonia and diarrhea, humanly euthanized due to the poor prognosis | Marked locally extensive pyogranulomatous pneumonia and pyogranulomas (*R. equi*; Mild to moderate neutrophilic hepatitis and enterocolitis |
| E25 | Yes | Severe gastric and esophageal ulceration, pyloric stricture-humanly euthanized | Moderate, chronic gastric ulceration and hyperkeratosis; Severe proximal duodenal stricture with gastric outflow obstruction; *Salmonella* culture positive |
| E26 | Yes | Presented with fever and hindlimb paresis, progressed coagulopathy with epistaxis, and died later. | Suppurative tubulointerstitial nephritis and bacterial septicemia (Klebsiella pneumoniae); Acute ulcerative colitis; Disseminated intravascular coagulation; Severe axonal degeneration (cervical, thoracic, lumbar spinal cord). |

**Supplementary Table 2**. List of Virulence gene primers used in this study.

| Gene | Primer Sequence (5’-3’) | Amplicon Size (bp) | Annealing Temp (^o^C) | References |
| --- | --- | --- | --- | --- |
| *invA* | F-CTCGCCTTTGCTCCTTTTAG | 211 | 58 | (1) |
|  | R-GCCATGGTATGGATTTGTCC |  |  |  |
| sipA | F-ATGGTTACAAGTGTAAGGACTCAG | 2055 | 55 | (2) |
|  | R-ACGCTGCATGTGCAAGCCATC |  |  |  |
| sipD | F-ATGCTTAATATTCAAAATTATTCCG | 1229 | 52 | (2) |
|  | R-TCCTTGCAGGAAGCTTTTG |  |  |  |
| sipC | F-cctggataatgactattgat | 300 | 55 | (3) |
|  | R-agtttatggtgattgcgtat |  |  |  |
| *avrA* | F- GGAAACCGATCTCGAAATGA | 241 | 53 | (4) |
|  | R- TGCTGGTTCGAACAAAATCA |  |  |  |
| *sopB* | F-GCTCTAGACCTCAAGACTCAAGATG | 1987 | 55 | (2) |
|  | R-GCGGCCGCTACGCAGGAGTAAATCGGTG |  |  |  |
| sopD | F-GAGCTCACGACCATTTGCGGCG | 1291 | 53 | (2) |
|  | R-GAGCTCCGAGACACGCTTCTTCG |  |  |  |
| *spvC* | F- ACTCCTTGCACAACCAAATGCGGA | 571 | 53 | (5) |
|  | R- TGTCTTCTGCATTTCGCCACC |  |  |  |
| sodC1 | F- CCAGTGGAGCAGGTTTATCG | 460 | 55 | (6) |
|  | R- GGTGCGCTCATCAGTTGTTC |  |  |  |
| *mgtC* | TGACTATCAATGCTCCAGTGAAT | 655 | 55 | (7) |
|  | ATTTACTGGCCGCTATGCTGTTG |  |  |  |
| *siiD* | GAATAGAAGACAAAGCGATCATC | 1231 | 55 | (8) |
|  | GCTTTGTCCACGCCTTTCATC |  |  |  |
| *fliC* | ACTGCTAAAACCACTACT | 366 | 52 | (9) |
|  | TGGAGACTTCGGTTGCGTAG |  |  |  |
| *csgA* | GCAATCGTATTCTCCGGTAG | 418 | 53 | (10) |
|  | GATGAGCGGTCGCGTTGTTA |  |  | (10) |
| *csgB* | F: TCCTGGTCTTCAGTAGCGTAA | 168 |  | (11) |
|  | R: TATGATGGAAGCGGATAAGAA |  |  |  |
| hilA | F-TTAAACATGTCGCCCAAACAGC | 216 | 55 | (12) |
|  | R- GCAAACTCCCGATGTAT |  |  |  |
| hilC | F-GGACTTGTTGCCAGGGATG | 241 | 63 | (12) |
|  | R-TGACCATTTGCGGGTGAG |  |  |  |

**Supplementary** **Table 3.** Breakpoints and Concentration of Antibiotics for Non typhoidal *Salmonella* according to CLSI 2024 guidelines.

| **Code** | **Antibiotics** | **Concentrations (μg/ml)** | **Breakpoints** | | |
| --- | --- | --- | --- | --- | --- |
|  |  |  | **S** | **I** | **R** |
| AK | Amikacin | 4-32 | ≤ 16 | 32 | ≥ 64 |
| AMP | Ampicillin | 0.25-32 | ≤ 8 | 16 | ≥ 32 |
| CAZ | Ceftazidime | 1-64 | ≤4 | 8 | ≥16 |
| XNL | Ceftiofur | 0.25-4 | ≤2 | 4 | ≥8 |
| CL | Chloramphenicol | 4-32 | ≤8 | 16 | ≥32 |
| DOX | Doxycycline | 2-16 | ≤4 | 8 | ≥16 |
| IMP | Imipenem | 1-8 | ≤1 | 2 | ≥4 |
| TE | Tetracycline | 2-8 | ≤4 | 8 | ≥16 |
| COT | Trimethoprim/sulfamethoxazole | 0.5/9.5-4/76 | ≤2/38 |  | ≥4/76 |
| GEN | Gentamicin | 1-8 | ≤4 | 8 | ≥16 |

**Supplementary Table 4**. List of AMR gene primers used in this study.

| Genes | | Primers | Length (bp) | Annealing Temp | Ref |
| --- | --- | --- | --- | --- | --- |
| β-lactams | *TEM* | F-ATGAGTATTCAACATTTCCG | 964 | 55 | (13) |
|  |  | R-ACCAATGCTTAATCAGTGAG |  |  |  |
|  | *CTX* | F-GAGTTTCCCCATTCCGTTTC | 909 | 55 | (13) |
|  |  | R-CAGAATAAGGAATCCCATGGTT |  |  |  |
|  | *SHV* | F-TTCGCCTGTGTATTATCTCCCTG | 854 | 53 | (13) |
|  |  | R-TTAGCGTTGCCAGTGCTCG |  |  |  |
|  | *OXA* | F-ACCAGATTCAACTTTCAA | 590 | 53 | (13, 14) |
|  |  | R-TCTTGGCTTTTATGCTTG |  |  |  |
| Tetracycline | *tetB* | F-GAGACGCAATCGAATTCGG | 228 | 52 | (13, 14) |
|  |  | R-TTTAGTGGCTATTCTTCCTGCC |  |  |  |
| Sulfonamides | *sul2* | F-GCGCTCAAGGCAGATGGCATT |  | 55 | (13) |
|  |  | R-GCGTTTGATACCGGCACCCGT | 285 |  |  |
| Chloramphenicol | *floR* | F-ATCCAACTCACGTTGAGCC | 868 | 52 | (13) |
|  |  | R-TTGGATGCAGAAGTAGAACG |  |  |  |
| Aminoglycosides | strA | F-CCAATCGCAGATAGAAGGC | 548 | 55 | (15) |
|  |  | R-CTTGGTGATAACGGCAATTC |  |  |  |
|  | *aacA(3)* | F- ATTGAAGATTTGCCAGAACA | 178 | 56.5 | (15, 16) |
|  |  | R- CACTATCATAACCACTACCG |  |  |  |
| Macrolide | erm (B) | F-GAAAAGGTACTCAACCAAATA | 639 | 52 | (16, 17) |
|  |  | R-GTAACGGTACTTAAATTGTTTAC |  |  |  |

**Supplementary Table 5.** Biofilm formation of *Salmonella* Isolates.

| Isolates | Serotypes | Biofilm Formation (OD_550_) | SD | Category |
| --- | --- | --- | --- | --- |
| E1 | Anatum | 0.3335 | 0.000707 | MBP |
| E2 | Enteritidiis | 0.234 | 0.034648 | MBP |
| E3 | Agbeni | 0.154 | 0.019799 | WBP |
| E4 | Thompson | 0.274 | 0.096167 | MBP |
| E5 | 4,(5),12:b:- | 0.185 | 0.014142 | WBP |
| E6 | 4,(5),12:b:- | 0.2005 | 0.027577 | WBP |
| E7 | Typhimurium | 0.179 | 0.011314 | WBP |
| E8 | 4,(5),12:i:- | 0.1915 | 0.027577 | WBP |
| E9 | Hartford | 0.2785 | 0.08556 | MBP |
| E10 | Typhimurium | 0.214 | 0.006364 | WBP |
| E11 | Thompson | 0.2565 | 0.051619 | MBP |
| E12 | Typhimurium | 0.1905 | 0.002121 | WBP |
| E13 | Anatum | 0.1585 | 0.014849 | WBP |
| E14 | Typhimurium | 0.154 | 0.022627 | WBP |
| E15 | Typhimurium | 0.183 | 0.009899 | WBP |
| E16 | Bovismorbificans | 0.243 | 0.05374 | MBP |
| E17 | Typhimurium | 0.117 | 0.002828 | WBP |
| E18 | Typhimurium | 0.1515 | 0.009192 | MBP |
| E19 | 4,[5],12:i:- | 0.2105 | 0.010607 | MBP |
| E20 | Typhimurium | 0.223 | 0.019799 | WBP |
| E21 | Hartford | 0.285 | 0.036062 | MBP |
| E22 | Hartford | 0.1595 | 0.017678 | WBP |
| E23 | Typhimurium | 0.1315 | 0.00495 | WBP |
| E24 | Anatum | 0.1455 | 0.00495 | WBP |
| E25 | Mbandaka | 0.539 | 0.083439 | SBP |
| E26 | Mbandaka | 0.4235 | 0.043134 | SBP |
| PC | Typhimurium | 0.449 | 0.046669 | SBP |

**Supplementary Table 6.** Swimming and Swarming motility of *Salmonella* isolates.

| **Isolates** | **Serotype** | **Swimming Motility (mm) ± SD** | **Swarming Motility (mm) ± SD** |
| --- | --- | --- | --- |
| E1 | Anatum | 3.8±0.2 | 2.8±0.2 |
| E2 | Enteritidiis | 3 | 2.8±0.2 |
| E3 | Agbeni | 3 | 2.6±0.2 |
| E4 | Thompson | 4 | 2 |
| E5 | 4,(5),12:b:- | 3 | 2.6±0.5 |
| E6 | 4,(5),12:b:- | 3 | 2.5 |
| E7 | Typhimurium | 5.8±0.2 | 2.5±0.5 |
| E8 | 4,(5),12:i:- | 4 | 3 |
| E9 | Hartford | 4 | 3 |
| E10 | Typhimurium | 3.6±0.5 | 2 |
| E11 | Thompson | 2.8±0.2 | 2.1±0.2 |
| E12 | Typhimurium | 6 | 2 |
| E13 | Anatum | 3.6±0.5 | 2.8±0.2 |
| E14 | Typhimurium | 4.5±0.5 | 3.5±0.5 |
| E15 | Typhimurium | 3.3±0.5 | 2.8±0.2 |
| E16 | Bovismorbificans | 4 | 2.8±0.2 |
| E17 | Typhimurium | 6 | 2.6±0.2 |
| E18 | Typhimurium | 5.3±0.5 | 2.3±0.5 |
| E19 | 4,[5],12:i:- | 5 | 3.3±0.2 |
| E20 | Typhimurium | 4.6±0.5 | 2.8±0.2 |
| E21 | Hartford | 3.6±0.5 | 3.6±0.5 |
| E22 | Hartford | 3 | 2.8±0.2 |
| E23 | Typhimurium | 3.8±0.2 | 2.1±0.2 |
| E24 | Anatum | 5.3±0.5 | 2 |
| E25 | Mbandaka | 3.6±0.5 | 2.8±0.2 |
| E26 | Mbandaka | 3 | 2 |

**Supplementary** **Table 7.** Antimicrobial resistance profile of the *Salmonella* isolates recovered from necropsied horses.

| Classes of Antimicrobials | Antimicrobials | %. of Salmonella isolates (No. of isolates) | | |
| --- | --- | --- | --- | --- |
|  | | **R** | **I** | **S** |
| Aminoglycosides | Gentamicin | 11.54 (3) | 0.00 (0) | 88.46 (23) |
|  | Amikacin | 11.54 (3) | 0.00 (0) | 88.46 (23) |
| Penicillin | Ampicillin | 11.54 (3) | 0.00 (0) | 88.46 (23) |
| Cephalosporins | Ceftazidime | 11.54 (3) | 0.00 (0) | 88.46 (23) |
|  | Ceftiofur | 11.54 (3) | 0.00 (0) | 88.46 (23) |
| Chloramphenicol | Chloramphenicol | 11.54 (3) | 0.00 (0) | 88.46 (23) |
| Carbapenems | Imipenem | 0.00 (0) | 0.00 (0) | 100.00 (26) |
| Tetracycline | Doxycycline | 0.00 (0) | 3.85 (1) | 96.15 (25) |
|  | Tetracycline | 3.85 (1) | 0.00 (0) | 96.15 (25) |
| Sulfonamides | Trimethoprim/Sulfamethoxazole | 11.54 (3) | 0.00 (0) | 88.46 (23) |

**Supplementary Table 8.** Prevalence of antimicrobial resistance genes within *Salmonella* isolates.

| **Category** | **Genes** | **Count** | **Prevalence (%)** | **Lower_CI (%)** | **Upper_CI (%)** | **p_value** |
| --- | --- | --- | --- | --- | --- | --- |
| Aminoglycosides | *strA* | 4 | 15.39 | 4.36 | 34.87 | 5.3E−4 |
|  | *aacA (3)* | 3 | 11.54 | 2.45 | 30.15 | 8.8E-05 |
| Aminoglycosides, Fluoroquinolones | *AAC(6')-Ib/Iaa* | 26 | 100 | 86.77 | 100 | 3E-08 |
| Beta-lactams | *blaTEM* | 3 | 11.54 | 2.45 | 30.15 | 8.8E-05 |
| Beta-lactams | *blaCTX-m* | 3 | 11.54 | 2.45 | 30.15 | 8.8E-05 |
|  | *bla SHV2* | 3 | 11.54 | 2.45 | 30.15 | 8.8E-05 |
|  | *blaOXA-9* | 1 | 3.85 | 0.10 | 19.64 | 8E-7 |
| Chloramphenicol, Florfenicol | *floR* | 3 | 11.54 | 2.45 | 30.15 | 8.8E-05 |
| Fluoroquinolones | *qnrB2* | 1 | 3.85 | 0.10 | 19.64 | 8E-7 |
|  | *gyrA* | 26 | 100 | 86.77 | 100 | 3E-08 |
| Macrolides, Lincosamides, Streptogramins | *ermB2* | 1 | 3.85 | 0.10 | 19.64 | 8E-7 |
| Sulfonamides | *sul2* | 5 | 19.23 | 6.55 | 39.35 | 2.5E-3 |
| Tetracyclines | *tetB* | 3 | 11.54 | 2.45 | 30.15 | 8.8E-05 |

**References**

1. Fazl AA, Salehi TZ, Jamshidian M, Amini K, Jangjou A. 2013. Molecular detection of invA, ssaP, sseC and pipB genes in Salmonella Typhimurium isolated from human and poultry in Iran. Afr J Microbiol ReS 7:1104-8.

2. Karacan Sever N, Akan M. 2019. Molecular analysis of virulence genes of Salmonella Infantis isolated from chickens and turkeys. Microbial Pathogenesis 126:199-204.

3. Hughes LA, Shopland S, Wigley P, Bradon H, Leatherbarrow AH, Williams NJ, Bennett M, De Pinna E, Lawson B, Cunningham AA. 2008. Characterisation of Salmonella enterica serotype Typhimurium isolates from wild birds in northern England from 2005–2006. BMC Veterinary Research 4:1-10.

4. Wang Y-P, Li L, Shen J-Z, Yang F-J, Wu Y-N. 2009. Quinolone-resistance in Salmonella is associated with decreased mRNA expression of virulence genes invA and avrA, growth and intracellular invasion and survival. Veterinary Microbiology 133:328-334.

5. Turki Y, Mehr I, Ouzari H, Khessairi A, Hassen A. 2014. Molecular typing, antibiotic resistance, virulence gene and biofilm formation of different Salmonella enterica serotypes. The Journal of General and Applied Microbiology 60:123-130.

6. Herrero A, Rodicio MR, González-Hevia MA, Mendoza MC. 2005. Molecular epidemiology of emergent multidrug-resistant Salmonella enterica serotype Typhimurium strains carrying the virulence resistance plasmid pUO-StVR2. Journal of Antimicrobial Chemotherapy 57:39-45.

7. Soto SM, Rodríguez I, Rodicio MR, Vila J, Mendoza MC. 2006. Detection of virulence determinants in clinical strains of Salmonella enterica serovar Enteritidis and mapping on macrorestriction profiles. Journal of medical microbiology 55:365-373.

8. Ren X, Li M, Xu C, Cui K, Feng Z, Fu Y, Zhang J, Liao M. 2016. Prevalence and molecular characterization of Salmonella enterica isolates throughout an integrated broiler supply chain in China. Epidemiology & Infection 144:2989-2999.

9. Ganesan V, Harish BN, Menezes GA, Parija SC. 2014. Detection of Salmonella in Blood by PCR using iroB gene. J Clin Diagn Res 8:Dc01-3.

10. Allen SE, Boerlin P, Janecko N, Lumsden JS, Barker IK, Pearl DL, Reid-Smith RJ, Jardine C. 2011. Antimicrobial resistance in generic Escherichia coli isolates from wild small mammals living in swine farm, residential, landfill, and natural environments in southern Ontario, Canada. Applied and environmental microbiology 77:882-888.

11. Rodrigues dos Santos EA, Ereno Tadielo L, Arruda Schmiedt J, Silva Orisio PH, de Cássia Lima Brugeff E, Sossai Possebon F, Olivia Pereira M, Gonçalves Pereira J, dos Santos Bersot L. 2023. Inhibitory effects of piperine and black pepper essential oil on multispecies biofilm formation by Listeria monocytogenes, Salmonella Typhimurium, and Pseudomonas aeruginosa. LWT 182:114851.

12. Rahn K, De Grandis S, Clarke R, McEwen S, Galan J, Ginocchio C, Curtiss Iii R, Gyles C. 1992. Amplification of an invA gene sequence of Salmonella typhimurium by polymerase chain reaction as a specific method of detection of Salmonella. Molecular and cellular probes 6:271-279.

13. Chen Z, Bai J, Wang S, Zhang X, Zhan Z, Shen H, Zhang H, Wen J, Gao Y, Liao M, Zhang J. 2020. Prevalence, Antimicrobial Resistance, Virulence Genes and Genetic Diversity of Salmonella Isolated from Retail Duck Meat in Southern China. Microorganisms 8.

14. Maravić A, Skočibušić M, Samanić I, Fredotović Z, Cvjetan S, Jutronić M, Puizina J. 2013. Aeromonas spp. simultaneously harbouring bla(CTX-M-15), bla(SHV-12), bla(PER-1) and bla(FOX-2), in wild-growing Mediterranean mussel (Mytilus galloprovincialis) from Adriatic Sea, Croatia. Int J Food Microbiol 166:301-8.

15. Asgharpour F, Mahmoud S, Marashi A, Moulana Z. 2018. Molecular detection of class 1, 2 and 3 integrons and some antimicrobial resistance genes in Salmonella Infantis isolates. Iran J Microbiol 10:104-110.

16. Gan T, Shu G, Fu H, Yan Q, Zhang W, Tang H, Yin L, Zhao L, Lin J. 2021. Antimicrobial resistance and genotyping of Staphylococcus aureus obtained from food animals in Sichuan Province, China. BMC Vet Res 17:177.

17. Ma X, Chen H, Wang F, Wang S, Wu Y, Ma X, Wei Y, Shao W, Zhao Y. 2023. Molecular characterisation and antimicrobial resistance of isolates from dairy farms in China. Journal of Veterinary Research 67:161-167.
